# Characterization of the Virome Associated with the Ubiquitous Two-Spotted Spider Mite, *Tetranychus urticae*

**DOI:** 10.3390/v16101532

**Published:** 2024-09-27

**Authors:** Lucas Yago Melo Ferreira, Anderson Gonçalves de Sousa, Joannan Lima Silva, João Pedro Nunes Santos, David Gabriel do Nascimento Souza, Lixsy Celeste Bernardez Orellana, Sabrina Ferreira de Santana, Lara Beatriz Correia Moreira de Vasconcelos, Anibal Ramadan Oliveira, Eric Roberto Guimarães Rocha Aguiar

**Affiliations:** 1 Center of Biotechnology and Genetics, Department of Biological Sciences, State University of Santa Cruz, Ilhéus 45662-900, Brazil; lucasmelobiomed@gmail.com (L.Y.M.F.); andersonbio93@gmail.com (A.G.d.S.); jlsilva.bio@uesc.br (J.L.S.); jpnsantos.bio@uesc.br (J.P.N.S.); davidgabriel-ns@hotmail.com (D.G.d.N.S.); lixsy09@gmail.com (L.C.B.O.); sabrinabiotec2@gmail.com (S.F.d.S.); laraseixas99@gmail.com (L.B.C.M.d.V.); 2 Laboratory of Entomology, Department of Biological Science, State University of Santa Cruz, Ilhéus 45662-900, Brazil; aoliveira@uesc.br; 3 Postgraduate Program in Computational Modeling in Science and Technology, Department of Engineering and Computing, State University of Santa Cruz (UESC), Ilhéus 45662-900, Brazil

**Keywords:** two-spotted spider mite, virome characterization, metatranscriptomics, RNA-seq

## Abstract

Agricultural pests can cause direct damage to crops, including chlorosis, loss of vigor, defoliation, and wilting. In addition, they can also indirectly damage plants, such as by transmitting pathogenic micro-organisms while feeding on plant tissues, affecting the productivity and quality of crops and interfering with agricultural production. Among the known arthropod pests, mites are highly prevalent in global agriculture, particularly those from the Tetranychidae family. The two-spotted spider mite, *Tetranychus urticae*, is especially notorious, infesting about 1600 plant species and causing significant agricultural losses. Despite its impact on agriculture, the virome of *T. urticae* is poorly characterized in the literature. This lack of knowledge is concerning, as these mites could potentially transmit plant-infecting viral pathogens, compromising food security and complicating integrated pest management efforts. Our study aimed to characterize the virome of the mite *T. urticae* by taking advantage of publicly available RNA deep sequencing libraries. A total of 30 libraries were selected, covering a wide range of geographic and sampling conditions. The library selection step included selecting 1 control library from each project in the NCBI SRA database (16 in total), in addition to the 14 unique libraries from a project containing field-collected mites. The analysis was conducted using an integrated de novo virus discovery bioinformatics pipeline developed by our group. This approach revealed 20 viral sequences, including 11 related to new viruses. Through phylogenetic analysis, eight of these were classified into the *Nodaviridae*, *Kitaviridae*, *Phenuiviridae*, *Rhabdoviridae*, *Birnaviridae*, and *Qinviridae* viral families, while three were characterized only at the order level within Picornavirales and Reovirales. The remaining nine viral sequences showed high similarity at the nucleotide level with known viral species, likely representing new strains of previously characterized viruses. Notably, these include the known *Bean common mosaic virus* (BCMV) and *Phaseolus vulgaris alphaendornavirus* 1, both of which have significant impacts on bean agriculture. Altogether, our results expand the virome associated with the ubiquitous mite pest *T. urticae* and highlight its potential role as a transmitter of important plant pathogens. Our data emphasize the importance of continuous virus surveillance for help in the preparedness of future emerging threats.

## 1. Introduction

Arthropods are a major agricultural pest, causing approximately 470 billion USD in crop production losses annually worldwide [1]. Among arthropods, mites (Arachnida: Acari) cause significant direct and indirect damage to cultivated plants, with some species contributing significantly to the rapid spread of diseases among different crops [2]. The family *Tetranychidae*, also known as the spider mite, stands out as one of the most significant pests affecting crops worldwide, as it induces chlorosis, loss of vigor, defoliation, and wilting of plants, and is considered a primary pest of several cultivated plants [3].

Tetranychid mites infest more than 4000 plant species, comprising a wide variety of commonly known and widely distributed classes [3,4]. While many of these mites are agricultural pests, only a few species within this family have been confirmed as carriers of plant viruses. For instance, the mite *Petrobia latens* has been identified as a vector for *Barley Yellow Mosaic Virus* (BaYSMV), primarily transmitted by *Polymyxa graminis* [5,6]. Likewise, *Tetranychus urticae* has been occasionally associated with the transmission of *Potato virus Y* (PVY), which is mainly vectored by aphids [7,8]. Additionally, *T. urticae* is known to vector several other viruses, including *Tobacco ringspot virus* (TRSV, primarily vectored by nematodes) [9], *Tobacco mosaic virus* (TMV, primarily transmitted mechanically) [10], *Southern bean mosaic virus* (SBMV, primarily vectored by beetles) [11], and *Cotton leaf curl virus* (CLCuV, primarily transmitted by whiteflies) [12]. Although *T. urticae* can acquire, carry, and excrete viruses during feeding, its ability to efficiently transmit these viruses is still poorly understood [13,14,15,16].

The two-spotted spider mite (*T. urticae*) is a major pest of various plant species worldwide [17,18,19,20,21,22], and several studies have investigated this mite for its biological cycle, reproductive parameters, population dynamics, survival probability, and specific fertility [23,24]. Understanding this aspects is crucial for the development of control strategies, especially as this species has been reported to affect about 1590 host plants [25]. However, the diversity of viruses infecting this significant crop pest, along with the plant pathogenic viruses it might carry, remains poorly characterized.

Here, we investigate the viral diversity of *T. urticae* by analyzing 30 publicly available RNA deep sequencing libraries. Our findings reveal a considerable diversity of new viruses and the presence of a known plant pathogenic virus, underscoring the importance of virus surveillance in spider mites, as their infestations can impact agriculture, thus posing a threat for human food security.

## 2. Materials and Methods

### 2.1. Acquisition of RNA Libraries

For this study, we conducted a comprehensive analysis to characterize the virome of the two-spotted spider mite *T. urticae*, using RNA-seq libraries available in the Sequence Read Archive (SRA) public database of the National Center for Biotechnology Information (NCBI). We selected a total of 30 libraries, covering a wide range of sampling conditions and geographical locations. This selection included one library of mites from a controlled laboratory environment from each RNA deep sequencing project available for *T. urticae* in the NCBI, in addition to fourteen libraries from the only two distinct projects containing field-collected mites. All 30 libraries underwent virus discovery pipeline analysis (Figure S1). Overall, these libraries were collected from *T. urticae* on different host plants, as well as field-collected mites on plants such as eggplant and bean. An overview of the libraries used can be found in Supplementary Table S1.

### 2.2. Identification of Endogenous Viral Elements

To distinguish between potential endogenous viral elements (EVEs) and exogenous virus species present in *T. urticae*, we screened its genome (GCF_000239435.1), which had been downloaded from the NCBI genome database. The identification process involved six steps: predicting open reading frames (ORFs), aligning sequences with a viral protein database, using Python for filtering and categorization, manually curating to remove unwanted sequences, clustering with CD-HIT (version 4.8.1) [26] to eliminate redundancy, and conducting a final alignment for validation. This pipeline methodology was previously detailed in Aguiar et al.(2020) [27]. An overview of the identified EVEs can be found in Supplementary Table S2.

### 2.3. Transcriptome Assembly

The analysis of public RNA-seq libraries was conducted using various bioinformatics tools, essentially following the protocol previously published by our group [28] (Figure S2). Briefly, raw reads were subjected to quality assessment using FastQC (version 0.74 + galaxy0) [29] to ensure data reliability. Subsequently, Trimmomatic (Galaxy Version 0.38.1) [30] was employed to remove low-quality reads (Phred < 20) and adapter sequences. To align the trimmed reads to the *T. urticae* genome (GCF_000239435.1), Bowtie2 (version 2.5.0 + galaxy0) [31] with default settings was used, enabling the identification and removal of host-related reads. The remaining unaligned reads were then subjected to the assembly process, utilizing SPAdes (version 3.15.4 + galaxy1) [32] with default settings.

### 2.4. Identification of Viral Sequences

To identify potential virus-derived contigs, a sequence similarity search was conducted using Diamond (2.0.15 + galaxy0) [33] in BlastX mode with an e-value threshold of 1 × 10^−5^, utilizing the NCBI Viral RefSeq database (release 218) as a reference. These analyses were performed on the Galaxy Australia platform [34].

### 2.5. Manual Curation of Viral Genomes

We removed retrotransposon-related contigs using an in-house Python script that processes the output from DIAMOND by first removing any sequences with “transposon”, “retroviral”, or “retrovirus” in the taxon name. Afterward, this script classifies all remaining sequences using the most updated ICTV master species list and the NCBI taxonomy through an API written in Python. The curated assembled contigs were filtered based on a minimum threshold of 1500 nucleotides. Subsequently, the non-redundant filtered sequences underwent manual analysis through BlastN and BlastX searches on the NCBI’s online platform. Contigs with hits showing coverage and identity greater than 90% were considered to be known virus species. Sequences with coverage greater than 20% and e-values lower than 1 × 10^−3^ for proteins and 1 × 10^−5^ for nucleotides were further analyzed. The ORFfinder software [35] was employed to predict ORFs within the sequences, while InterProscan [36] and CD-Search [37] were used to identify the presence of viral conserved domains. Contigs that lacked conserved domains similar to their best hits were excluded for further analyses. A summary of the similarity search results obtained at the nucleotide and amino acid levels for each viral sequence can be found in Supplementary Table S3.

### 2.6. Phylogenetic Analysis

Sequences showing similarity to genes encoding RNA polymerase or polyproteins were used for constructing phylogenetic trees. Additionally, protein sequences representing specific families, such as *Dicistroviridae*, *Nodaviridae*, *Virgaviridae*, *Rhabdoviridae*, *Phenuiviridae*, *Birnaviridae*, *Qinviridae* and others within the orders Picornavirales and Reovirales, were included based on proximity and references from the International Committee on Taxonomy of Viruses (ICTV). For each set of sequences, a global alignment was performed using the MAFFT online platform [38] to ensure accurate sequence alignment. Subsequently, the IQ-TREE version 2.0.3 program [39] was used on a local server to generate a maximum likelihood phylogenetic tree, with 1000 bootstrap replicates to assess the robustness of the tree topology. The phylogenetic tree was then edited using Inkscape version 1.4.4 [40].

### 2.7. Transcriptional Activity of Viral Sequences

The transcriptional activity of virus-derived sequences was assessed using the Salmon software (version 0.14.1) [41]. For comparison with viral abundance, the host mitochondrial ribosomal protein L13 (rpl13) and nuclear calmodulin-1 (calmodulin) genes were selected as endogenous and standard references, respectively. An overview of the transcriptional activity of the viral sequences used in this analysis can be found in Supplementary Table S4.

## 3. Results

### 3.1. Characterization of Endogenous Viral Elements

The study of endogenous viral elements (EVEs) integrated into the *T. urticae* genome revealed two distinct sequences, 1032 nt and 1239 nt in length, which were identified through similarity searches using blastX. These EVEs showed significant similarities to nucleocapsid and nucleoprotein from elements within *Rhabdoviridae* family (see Supplementary Table S2 for details). Interestingly, both sequences showed large ORFs and presented the conserved domain Rhabdo_ncapsid, which could mislead the identification of exogenous viral sequences. Therefore, these sequences were used as negative controls for the identification of putative exogenous sequences.

### 3.2. Virome Characterization

**Figure 1:**
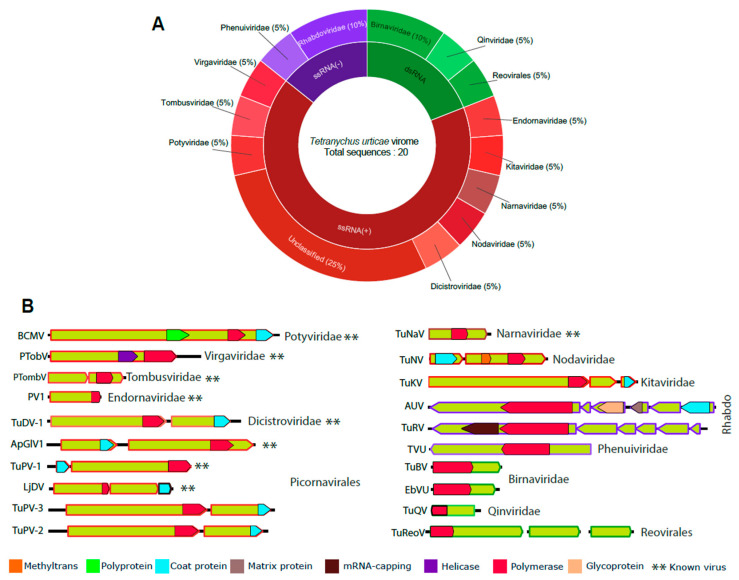
Diversity and genomic characterization of *Tetranychus urticae*-associated viruses. (**A**) Sunburst plot representing the viral diversity (20 sequences) characterized in *T. urticae*. The inner ring represents the viral genome composition, followed by the representation of the viral taxons to which the sequences belong. In total, 20 sequences were found, including Picornavirales: 5; *Dicistroviridae*: 1; *Potyviridae*: 1; *Virgaviridae*: 1; *Tombusviridae*: 1; *Endornaviridae*: 1; *Narnaviridae*: 1; *Nodaviridae*: 1; *Kitaviridae*: 1; *Rhabdoviridae*: 2; *Phenuiviridae*: 1; *Birnaviridae*: 2; *Qinviridae*: 1; and Reovirales: 1. (**B**) Genomic characteristics and conserved domains of the identified viral sequences. The sequence length is determined by the line extension, while the open reading frames (ORFs) are represented by the green box and conserved domains in different colors according to its putative function. The identified viruses include the following: *Bean common mosaic virus* (BCMV), Plant-associated tobamo-like virus 1 (PTobV), Plant-associated tombus-like virus 2 (PTombV), *Phaseolus vulgaris alphaendornavirus 1* (PV1), *Aphis glycines virus 1* (ApGlV1), *Ljubljana dicistrovirus 1* (LjDV), Tetranychus urticae picorna-like virus 1-3 (TuPV-1-3), Tetranychus urticae dicistro-like virus 1 (TuDV-1), Tetranychus urticae-associated narnavirus (TuNaV), Tetranychus urticae noda-like virus (TuNV), Tetranychus urticae kita-like virus (TuKV), Tetranychus urticae rhabdo-like virus (TuRV), Alphapaprhavirus urticae (AUV), Tetranychus urticae birnavirus (TuBV), Entomobirnavirus urticae (EbVU), Tetranychus urticae qin-like virus (TuQV) and Tetranychus urticae reo-like virus (TuReoV). Previously characterized viral species are indicated by (**).

Metatranscriptomic analysis revealed a wide spectrum of viral sequences, leading us to conduct a more detailed investigation of the samples to determine the presence of exogenous viruses. Therefore, we performed comprehensive sequence similarity searches against non-redundant nucleotide and protein databases of NCBI. This analysis identified several putative viral sequences, including positive single-stranded RNA viruses (ssRNA+), negative single-stranded RNA viruses (ssRNA−), and double-stranded RNA viruses (dsRNA) (Figure 1A and Supplementary Table S3).

At the family level, the ssRNA(+) genomes included representatives from the families *Tobamoviridae*, *Tombusviridae*, *Potyviridae*, *Dicistroviridae*, *Narnaviridae*, *Nodaviridae*, *Kitaviridae*, and others within the order Picornavirales. For the ssRNA(−) genomes, elements from the families *Rhabdoviridae* and *Phenuiviridae* were observed. The dsRNA viral viruses identified are related to elements from *Birnaviridae* and *Qinviridae* families and the Reovirales order (Figure 1A and Supplementary Table S3).

#### 3.2.1. Characterization of Known Viruses

Nine of the assembled contigs showed sequence similarity to previously characterized viral species of greater than 90% at both the nucleotide and amino acid levels. The first contig, 10,013 nt long, presented a complete polyprotein ORF of 9609 nt. This ORF displayed the same domains as the potyvirus *Bean common mosaic virus* (BCMV), its closest hit. The domains included ps-ssRNAv_Potyviridae_RdRp, the Poty_coat superfamily, and the Poty_PP superfamily (Figure 1B and Figure S3). Accordingly, this sequence was identified as a strain of the BCMV. The second contig, 6603 nt long, is related to an unclassified tobamovirus, encoding to a large ORF of 5436 nt and presenting the same domains as its closest match, Plant-associated tobamo-like virus 1 (PTobV), including the Virgaviridae_RdRp and Helicase domains (Figure S3). The third contig, 3348 nt long, contained two ORFs of 1686 and 1458 nt, and displayed the same domains as its closest hit, Plant associated tombus-like virus 2 (PTombV), including the Tombusviridae_RdRp domain (Figure 1B and Figure S3).

A narnavirus-related contig, 2704 nucleotides in length, showed over 90% nucleotide identity with its closest match, the Tetranychus urticae-associated narnavirus. This contig contains a 2487-nucleotide ORF that encodes only the ps-ssRNAv_Narnaviridae_RdRp domain (Figure 1B and Figure S3). Another contig displayed a high nucleotide identity of 99.82% to *Phaseolus vulgaris alphaendornavirus* 1 (PV1). This contig, 1623 nucleotides long, contains an incomplete 1503-nucleotide ORF and features the Endornaviridae_RdRp domain (Figure 1B and Figure S3).

The remaining four contigs, ranging from 2704 to 9009 nucleotides in length, showed over 90% nucleotide identity to known picornaviruses, including Tetranychus urticae-associated dicistrovirus 1 (TuDV-1), Tetranychus urticae-associated picorna-like virus 1 and (TuPV-1), Aphis glycines virus 1 (ApGlV1), and Ljubljana dicistrovirus 1 (LjDV) (Figure 1B and Figure S3). All contigs contained two complete ORFs, featuring the ps-ssRNAv-Picornavirales and coat protein domains (Figure 1B and Figure S3). These viruses were subsequently classified as new strains of their closest matches and were not included in the phylogenetic analysis.

#### 3.2.2. Characterization of Novel Viruses

##### Order Picornavirales

**Figure 2:**
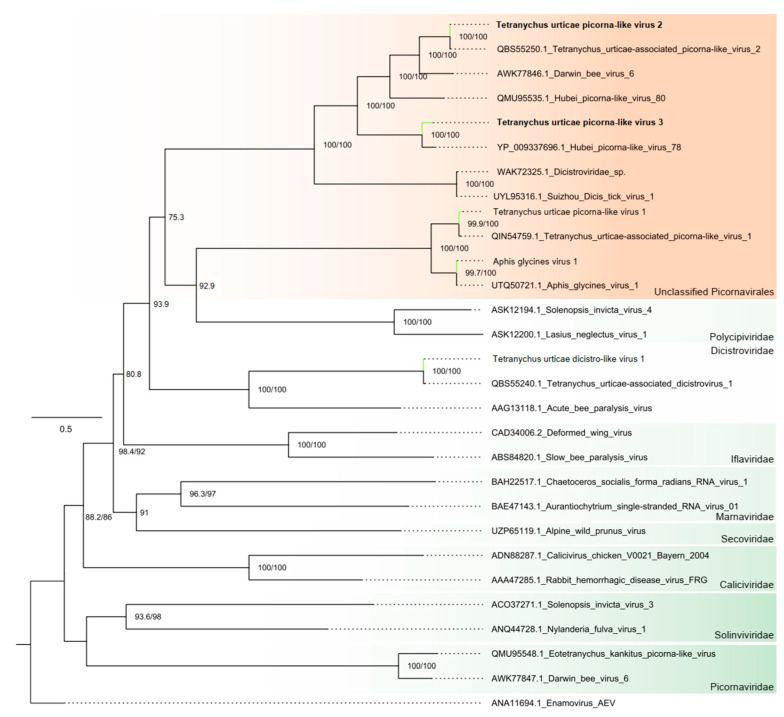
Phylogenetic reconstruction of assembled sequences related to the Picornavirales order: Maximum likelihood phylogenetic tree constructed using full-length amino acid sequences of the RNA-dependent RNA polymerase (RdRp). The tree was generated using IQ-TREE with the LG+F+R4 evolutionary model and 1000 bootstrap pseudoreplicates. To root the tree, one sequence of ssRNA+ Enamovirus from the *Solemoviridae* family was used as outgroup.

Two assembled contigs showed amino acid-level similarities with members of the order Picornavirales. The contigs, 9495 and 9770 nt long, represented complete coding sequences (two ORFs) according to the closest viral species. The larger ORF displayed conserved polymerase domains, whereas the second ORF displayed conserved capsid-related domains typical of picornaviruses. These contigs had the same conserved domains as their closest viruses, as detailed in Figure 1B and Figure S3. The polymerases were subjected to phylogenetic analysis, and the two contigs clustered within unclassified Picornavirales viruses (Figure 2). They were subsequently named Tetranychus urticae picorna-like virus 2 (TuPV-2) and Tetranychus urticae picorna-like virus 3 (TuPV-3).

##### Nodaviridae

**Figure 3:**
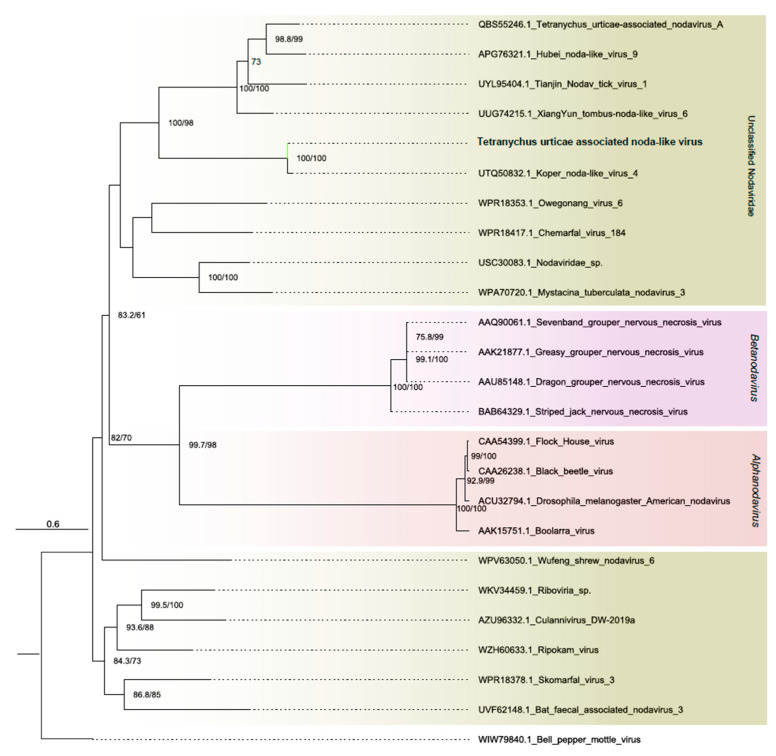
Phylogenetic analysis of nodavirus-related sequence: Maximum likelihood phylogenetic tree constructed using full-length amino acid sequences of the RNA-dependent RNA polymerase (RdRp). The tree was generated using IQ-TREE with the LG+F+I+G4 evolutionary model and 1000 bootstrap pseudoreplicates. To root the tree, sequence of ssRNA+ *Bell pepper mottle virus* from the *Virgaviridae* family was used as outgroup.

One assembled contig showed amino acid-level similarity with members of the *Nodaviridae* family. Spanning 5091 nt, the sequence presented two complete ORFs. The first, 3435 nt long, displayed three conserved domains: RNA-directed RNA polymerase catalytic domain (IPR007094), RNA-directed RNA polymerase C-terminal domain (IPR001205), and Nodavirus methyltransferase domain (IPR043647), overlapping the homologous DNA/RNA polymerase superfamily (IPR043502). The second ORF, 1410 nt long, showed only one homologous superfamily, viral coat protein subunit (IPR029053) (Figure 1B and Figure S3). Phylogenetic analysis grouped the assembled sequence with unclassified elements from the *Nodaviridae* family. Therefore, this sequence was named Tetranychus urticae noda-like virus TuNV(Figure 3).

##### Kitaviridae

**Figure 4:**
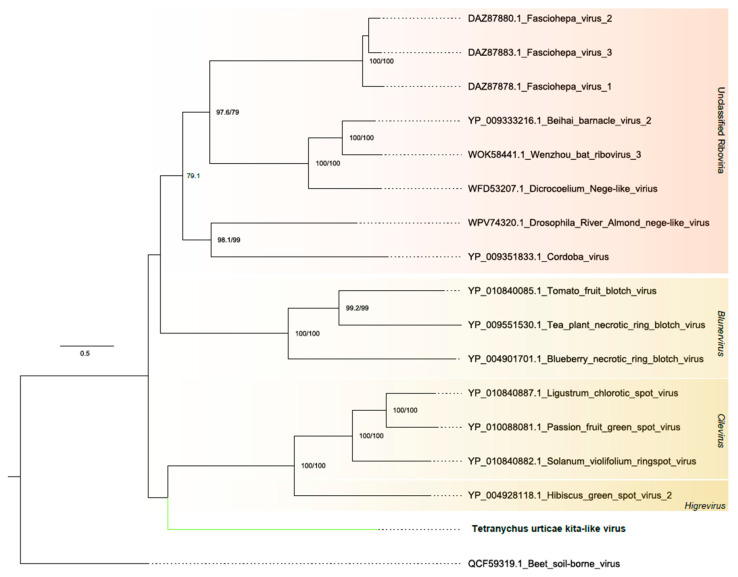
Phylogenetic analysis of kitavirus-related sequence: Maximum likelihood phylogenetic tree constructed using full-length amino acid sequences of the RNA-dependent RNA polymerase (RdRp). The tree was generated using IQ-TREE with the LG+F+R5 evolutionary model and 1000 bootstrap pseudoreplicates. To root the tree, one sequence of ssRNA+ *Middelburg virus* from the *Togaviridae* family was used as outgroup.

One assembled contig was related to members of the *Kitaviridae* family. The contig, spanning 9052 nt, exhibited three complete and large ORFs. The first of these, which was 6963 nt long, showed two conserved domains: RNA-dependent RNA polymerase alsuviricetes (IPR001788) and RNA-directed RNA polymerase catalytic domain (IPR007094), overlaid with the homologous DNA/RNA polymerase superfamily (IPR043502). The second, with a length of 1134 nt, encodes to coat protein domains, while the third did not present conserved domains (Figure 1B and Figure S3). Phylogenetic analysis showed that this sequence clustered with unclassified kitaviruses (Figure 4). Accordingly, this virus was named Tetranychus urticae kita-like virus (TuKV).

##### Rhabdoviridae

**Figure 5:**
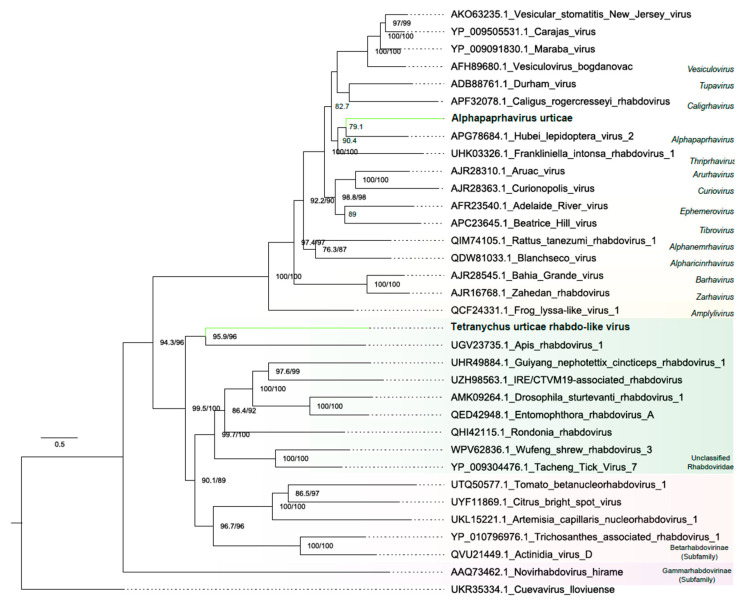
Phylogenetic analysis of rhabdoviral sequences: Maximum likelihood phylogenetic tree constructed using full-length amino acid sequences of the RNA-dependent RNA polymerase (RdRp). The tree was generated using IQ-TREE with the LG+F+R7 evolutionary model and 1000 bootstrap pseudoreplicates. To root the tree, one sequence of *Cuevavirus lloviuense* from the *Filoviridae* family was used as outgroup.

Two assembled contigs showed similarity at the amino acid level with members of the *Rhabdoviridae* family (Figure 1 and Figure S3). The first contig, 12,459 nt long, primarily consisted of five large ORFs ranging from 753 to 6474 nt. It exhibited conserved Rhabdovirus-related domains, including the Mononeg_RNA_pol superfamily, the Rhabdo_glycop superfamily, the Rhabdo_ncap superfamily, and the Vesiculo_matrix superfamily (Figure 1B). The genomic structure of the viruses resembled the known virus, Vesicular stomatitis New Jersey virus (Figure S3). Use of the RdRp, phylogenetic analysis indicated that the virus is closely related to unclassified *Rhabdoviridae* viruses (Figure 5).

The second contig, 12,072 nt long, encodes to five large ORFs ranging from 813 to 6213 nt. Similar to the previous sequence, this contig presented Rhabdovirus-related conserved domains, such as the Mononeg_RNA_pol superfamily, the Mononeg_mRNAcap superfamily, the paramyx_RNAcap superfamily, and the Rhabdo_ncap_2 superfamily (Figure 1B). Phylogenetic analysis grouped the virus within a group of unclassified *Rhabdoviridae* viruses (Figure 5). These new viruses were named as Tetranychus urticae rhabdo-like virus (TuRV) and Alphapaprhavirus urticae (AUV).

##### Phenuiviridae

**Figure 6:**
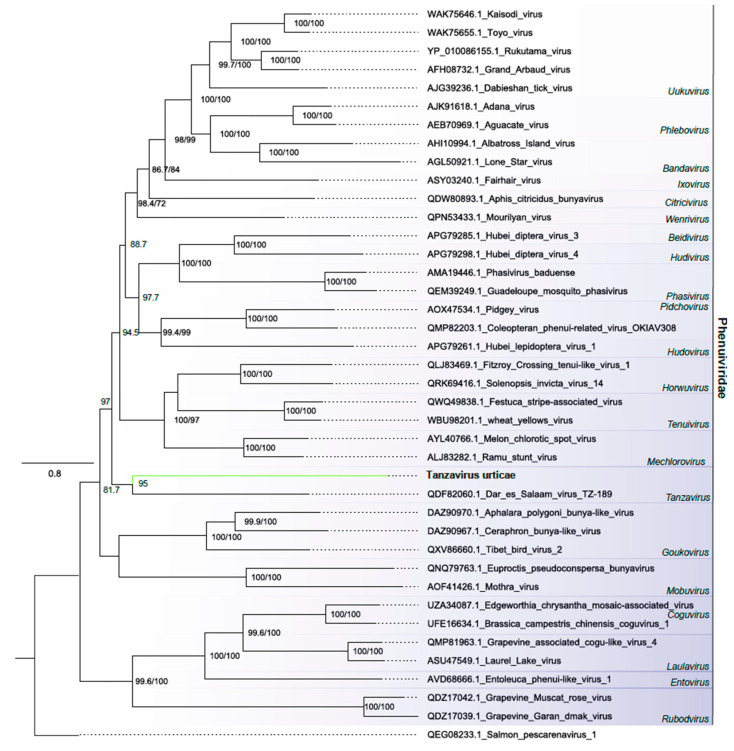
Phylogenetic analysis of the phenuivirus-related sequence: Maximum likelihood phylogenetic tree constructed using full-length amino acid sequences of the RNA-dependent RNA polymerase (RdRp). The tree was generated using IQ-TREE with the LG+F+R6 evolutionary model and 1000 bootstrap pseudoreplicates. To root the tree, one sequence of Salmon pescarenavirus 1 from the *Arenaviridae* family was used as outgroup.

We identified one contig related to members of the *Phenuiviridae* family (Figure 1B and Figure S3). This family is composed of negative stranded three-segmented viruses encoding the proteins L, which encodes the viral RNA polymerase; glycoproteins; and the proteins S, M and nucleocapsid (N) [42]. However, in this analysis, we only found the segment linked to RdRp. The contig, 7514 nt long, exhibited a complete ORF of 7440 nt encoding to RNA-directed RNA polymerase, negative-strand RNA virus (IPR007099), RNA-directed RNA polymerase L, N-terminal (IPR029124), and RNA-dependent RNA polymerase, bunyaviral (IPR007322) domains. Phylogenetic analysis revealed that the assembled viral sequence clustered with elements classified in the Tanzavirus genus within the *Phenuiviridae* family (Figure 6). This viral sequence was named Tanzavirus urticae (TVU).

##### Birnaviridae

**Figure 7:**
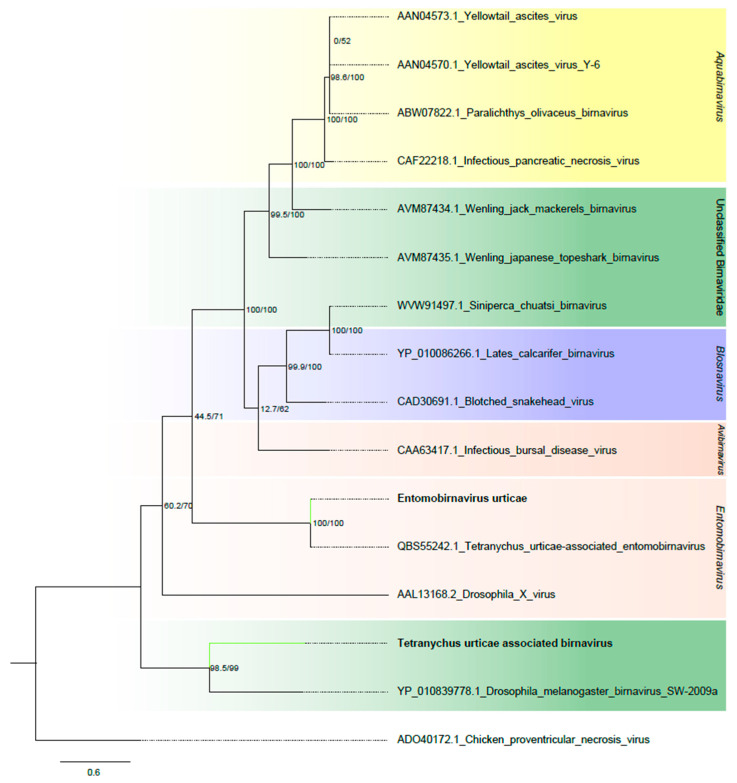
Phylogenetic analysis of birnavirus-related sequences: Maximum likelihood phylogenetic tree constructed using full-length amino acid sequences of the RNA-dependent RNA polymerase (RdRp). The tree was generated using IQ-TREE with the LG+I+G4 evolutionary model and 1000 bootstrap pseudoreplicates. To root the tree, one sequence of *Chicken proventricular necrosis virus,* an unclassified birnavirus, was used as outgroup.

Two contigs, one 3061 and the other 2950 nt long, were found to be related to members of the *Birnaviridae* family, which are composed of two genomic segments: Segment A, encoding five to six proteins and Segment B, encoding the RdRp [43]. Unfortunately, we only identified the RdRp segment (Segment B) for both putative viruses. The two contigs contained complete ORFs of 2892 nt and 2646 nt, respectively. They displayed the Birna_RdRp_palm superfamily conserved domains, resembling their closest matches, *Drosophila melanogaster birnavirus* and Tetranychus urticae-associated entomobirnavirus (Figure 1B and Figure S3). In phylogenetic analysis, one viral sequence clustered with unclassified members of the *Birnaviridae* family, while the other grouped with viruses within the Entomobirnavirus genus (Figure 7). Accordingly, they were named Tetranychus urticae-associated birnavirus (TuBV) and Entomobirnavirus urticae (EbVU).

##### Qinviridae

**Figure 8:**
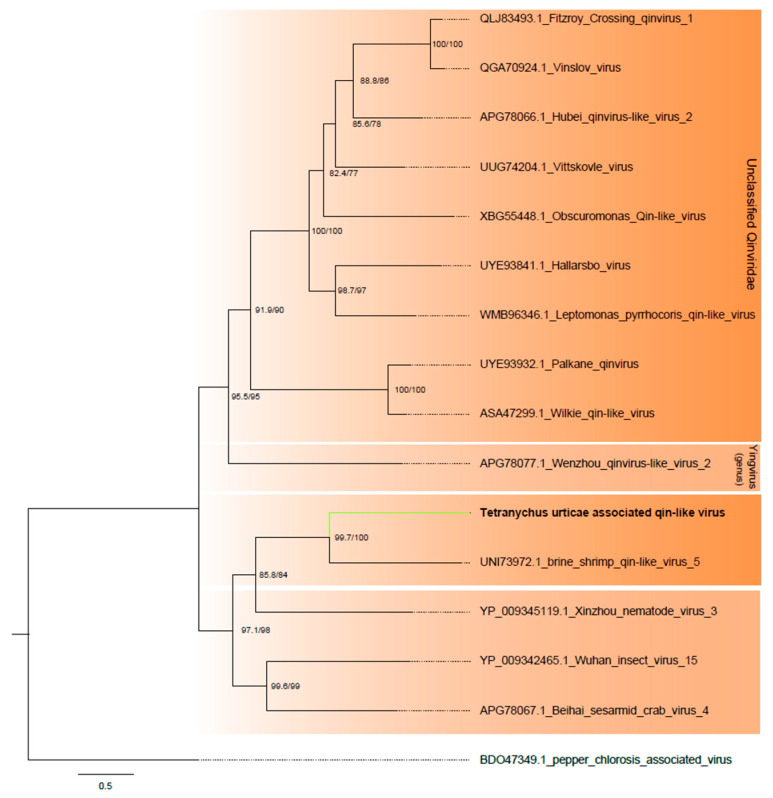
Phylogenetic analysis of the qin virus-related sequence: Maximum likelihood phylogenetic tree constructed using full-length amino acid sequences of the RNA-dependent RNA polymerase (RdRp). The tree was generated using IQ-TREE with the LG+F+R4 evolutionary model and 1000 bootstrap pseudoreplicates. To root the tree, one sequence of pepper chlorosis-associated virus from the family *Aspiviridae* was used as outgroup.

One of the assembled contigs showed sequence similarity at the amino acid level with viruses within the *Qinviridae* family. The contig, 2163 nt long, contained an incomplete ORF of 1875 nt, encoding to a Mononeg_RNA_pol superfamily domain (Figure 1B and Figure S3). Phylogenetic analysis indicated that the sequence clustered with unclassified *Qinviridae* viruses (Figure 8). The viral species was named Tetranychus urticae-associated qin-like virus (TuQV).

##### Reovirales

**Figure 9:**
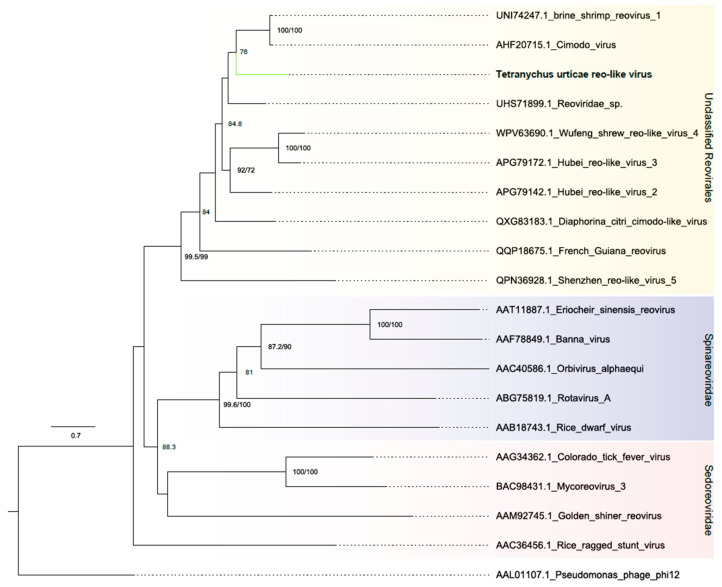
Phylogenetic analysis of reovirus-related sequence: Maximum likelihood phylogenetic tree constructed using full-length amino acid sequences of the RNA-dependent RNA polymerase (RdRp). The tree was generated using IQ-TREE with the LG+F+R5 evolutionary model and 1000 bootstrap pseudoreplicates. To root the tree, one sequence of *Pseudomonas phage phi12* from the *Cystoviridae* family was used as an outgroup.

The last three contigs were related to viruses within the Reovirales order, which consists of nonenveloped viruses with dsRNA genomes comprising 9–12 segments [44]. However, in this analysis we detected only three complete segments. One segment was 4194 nt long and contained a complete ORF of 3969 nt that exhibited the RdRP-N (IPR054006) domain. The other two segments displayed sequence similarity to hypothetical protein and NTP-binding domain proteins of reoviruses, but did not contain conserved domains, similar to their best hits (Figure 1B and Figure S3). Phylogenetic analysis based on the RdRp protein grouped the sequence with unclassified Reovirales viruses (Figure 9). The putative virus was named Tetranychus urticae-associated reo-like virus (TuRV).

### 3.3. Transcriptional Activity and Widespread of Viral Sequences

**Figure 10:**
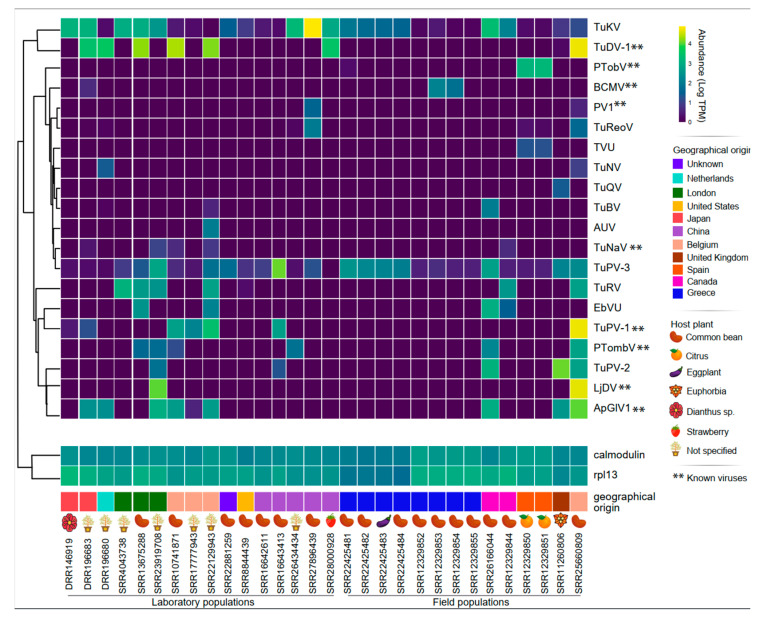
Assessment of the transcriptional activity of *Tetranychus urticae*-associated viruses. Heatmap representing the transcriptional activity of identified viral sequences in *T. urticae*-derived samples. The color spectrum reflects transcription levels, ranging from high (yellow) to low (blue). The heatmap employs row clusterization based on Pearson correlation to group sequences with similar profiles. The abundance was normalized by transcripts per million (TPM) and plotted in log10 scale. Samples are separated by laboratory populations and field populations with respect to geographical location and the host plant from which mites were collected.

After characterizing the putative new viral sequences, we determined their transcriptional activity by quantifying the viral transcripts alongside constitutive host marker genes. Most of the examined sequences exhibited transcriptional activity with equal or higher abundance than the constitutive genes. Notably, all sequences displayed transcriptional activity in at least two distinct libraries, except for TuQV and AUV. The first virus was only detected in a field-collected sample from the United Kingdom, while AUV was only found in a sample from controlled environment population from Belgium (Figure 10).

The TuDV-1, TuKV, TuPV-1, and Ljubljana viruses showed abundances 10–20 times higher than the constitutive genes, while the TuNaV, TuNV, and TuQV viruses showed RNA levels lower than the host calmodulin and rpl13 genes. It is important to note that the TuKV, TuDV-1, TuRV, ApGlV1 and TuPV-3 viruses showed transcriptional activity in distinct libraries from different projects, locations, and sample conditions, which indicates that they are likely components of the *T. urticae* resident virome (Figure 10).

After evaluating the abundance of *T. urticae*-associated viruses, we analyzed the RNA coverage profile for the identified BCMV and PV1 viral sequences, due to their agricultural importance. Our findings reveal that both viruses displayed coverage from both genomic and antigenomic strands along their entire length (Figure S4). Two Belgian samples exhibited the highest viral species diversity in the study, both in field and laboratory populations. In contrast, the samples with the least viral diversity were from field populations in Greece and laboratory populations in China and Belgium (Figure 10).

Regarding the viral sequence distribution, a higher number of viral sequences were found within libraries from mite control populations, while libraries from mite field populations contained fewer viral sequences. Despite this subtle difference, the viruses present in both populations depicted a similar abundance (Figure 10).

## 4. Discussion

Metatranscriptomics has become an important scientific approach aimed at unraveling the complex ecosystems that drive life on the planet, significantly contributing to the current understanding of the interaction between micro-organisms and their hosts. Considering the study of the two-spotted spider mite, *Tetranychus urticae*, an important agricultural pest, the metatranscriptomic analysis of RNA-seq data obtained from 30 publicly available libraries in the NCBI shed light on the virome of this mite in different countries, plant hosts and under various conditions. Surprisingly, the viral diversity that was found encompasses a wide variety of RNA viruses, including families often associated with plant pathogenesis.

As a result of this approach, and with regard to the known viral species from plant-infecting viral families, three unclassified virus members of the *Virgaviridae*, *Tombusviridae* and *Endornaviridae* families were identified, along with the potyvirus *Bean common mosaic virus* (BCMV), which is a major bean pathogen that can cause up to 80% yield loss. These viral families assume significant importance due to their diversified impact on a variety of economically valuable crops, including beans, tomatoes, peanuts, cucumbers, peas, and several others [45,46,47,48].

Expanding on the topic, the common bean, the host of BCMV [49] and PV1 [50], is among the best-known legume crops in human nutrition, with more than 300 million people worldwide dependent on them as a primary source of dietary protein. In 2015, the estimated harvested area of bean crops was 27 million hectares, producing nearly 29 million tons of dry beans [51,52]. BCMV-infected beans can experience yield losses of up to 80% [53]. Crops such as beans, potatoes, tomatoes, cucumbers, peanuts, and others are highly susceptible to diseases caused by viruses [45,48,54]. The most significant symptom of virus infections in these plants is the yellowing of leaves or the mosaic pattern of light and dark green shades, along with wilting, fruit drying, and plant death [55]. It is also important to note that viruses within the *Potyviridae*, BCMV viral family have been reported to be transmitted by mites [7,56,57]. PV1 is a persistent virus [50]. Unlike acute viral infections that typically cause pronounced symptoms and can be lethal to the host [58], persistent viruses like PV1 establish long-term associations with their hosts [59]. The complete effects of PV1 on its host are not fully elucidated, but some studies suggest that PV1 may have potential beneficial effects and has been associated with improved seed germination and increased seed weight, which could confer an advantage to infected plants in terms of reproductive success and competitive ability [60]. Additionally, persistent endornaviruses have not been reported to be transmitted horizontally [59,61].

Regarding the known mite-borne viral species, TuPV-1 is emerging as a candidate because it is part of the resident virome of *T. urticae*. The detection of TuPV-1 in distinct *T. urticae* populations from geographically separate locations further supports the hypothesis that this virus is consistently associated with this species [55]. However, the current data are insufficient to conclusively define its position as a resident virus, and further investigation is necessary to clarify the potential evolutionary relationships among this virus and mites.

Among the newly identified viral species, some were classified within the Picornavirales order. These viruses typically possess monocistronic genomes, containing a single open reading frame that encodes a large polyprotein. However, the *Dicistroviridae* family is an exception; its RNA genome is dicistronic, featuring two non-overlapping ORFs separated by an intergenic untranslated region (IGR). The first ORF encodes replication proteins, while the second ORF encodes structural proteins. These characteristics were also observed in the TuPV-2 and TuPV-3 viruses identified in this study. Notably, viruses in the Picornavirales order are primarily known for their affinity with invertebrates (*Dicistroviridae* and *Iflaviridae*), vertebrates and plants (*Secoviridae* and *Picornaviridae*) and algae (*Marnaviridae*). This unique characteristic has sparked interest in the potential of some families in terms of their application to the development of biological control strategies aimed at managing populations of insect pests [62].

TuNV is similar to members of the *Nodaviridae*, which is a family of small, non-enveloped viruses with bipartite genomes of ssRNA (+). The virions are non-enveloped and have a spherical shape. The genome consists of two molecules of ssRNA (+), RNA1 and RNA2. RNA1 encodes protein A, an RNA-dependent RNA polymerase, while RNA2 encodes protein α, the precursor of the capsid protein. Alphanodaviruses infect insects, while betanodaviruses are pathogens of fish [63,64].

The viruses of the *Kitaviridae* family are characterized by their ssRNA(+), typically ranging in size from 6 to 8 kb. These genomes are organized so as to encode a replication-associated protein and a coat protein. It is known that crops infected by this virus tend to develop non-systemic diseases where only locally infected tissues show atypical chlorotic and/or necrotic lesions [65,66], which affect the aesthetics of the product and consequently its sale. In the virome of *T. urticae* (Acari, Tetranychidae), our results show the presence of a new virus with transcriptional activity, belonging to the *Kitaviridae* family, which has not yet been classified, and which has been named Tetranychus urticae kita-like virus (TuKV), in mites that were collected in the field and in the laboratory. This result is relevant because the viruses of the *Kitaviridae* family that infect plants of economic importance, such as citrus, tomato, passion fruit, tea and blueberry, are transmitted by eriophid mites and some species of mites of the genus Brevipalpus (Acari, Tenuipalpidae) in a persistent way, which means that once a mite acquires the virus, it can transmit it for the rest of its life [67,68].

Tetranychus urticae- rhabdo-like virus (TuRV) and Alphapaprhavirus urticae (AUV) were classified into the *Rhabdoviridae* family, which ecologically consists of a diverse group of viruses that infect terrestrial and aquatic vertebrates, invertebrates, and plants. They include many pathogens of importance to public health, agriculture, and fisheries [69]. Additionally, they primarily contain non-segmented negative-sense single-stranded RNA (ssRNA−) genomes, with lengths ranging 11–16 kb. The basic genome organization shared by all rhabdoviruses includes five canonical genes encoding (3′ to 5′) the nucleoprotein (or nucleocapsid protein, N), the phosphoprotein (P), the matrix protein (M), the glycoprotein (G), and the large protein (L, RNA-dependent RNA polymerase) [69]. Studies that have included host genome mass sequencing have revealed the integration of rhabdovirus-like elements into the genomes of some arthropods and plants, suggesting an ancient evolutionary origin and a long-standing association of rhabdoviruses with their hosts [70]. Indeed, these findings correlate with the endogenous and exogenous rhabdoviruses’ viral sequences found in *T. urticae*.

The *Phenuiviridae* family harbors viruses that infect three kingdoms of host organisms: animals, plants, and fungi, which is rare among known viral families [71]. Multiple phenuiviruses are highly pathogenic to humans, animals, or plants [63]. They impose heavy global burdens on human health, the livestock industry, and agriculture. Phenuiviruses are characterized by their segmented (ssRNA−) genome. This genome is organized into three distinct segments, each with specific functions. Segment L encodes the RdRp and is about 6.4 kb in length. Segment M encodes the glycoprotein and is about 3.2 kb in length. Lastly, segment S encodes the nucleocapsid protein and is about 1.7 kb in length [42]. Our Tanzavirus urticae virus shares similar structural and genetic characteristics with *Phenuiviridae*.

*Birnaviridae* and *Qinviridae* are two distinct families of non-enveloped, bi-segmented double-stranded RNA viruses that typically infect invertebrates [43,72]. The order Reovirales, on the other hand, encompasses a wider variety of viruses with more diverse genome structures ranging from 9 to 12 segments. This segmented nature, a hallmark of Reovirales, allows for genetic reassortment during co-infection, potentially leading to the emergence of new viral strains [73]. The Tetranychus urticae-associated birnavirus, Entomobirnavirus urticae, Tetranychus urticae qin-like virus and Tetranychus urticae reo-like virus viruses found in our study share the same characteristics as the *Birnaviridae* and *Qinviridae* families, and the Reovirales order.

We meticulously investigated the transcriptional activity of various sequences and, interestingly, all viruses displayed signs of transcriptional activity. Except for TuQV and TuRV-2, found only in one library, each sequence was identified in a minimum of two libraries, with the TuDV-1, TuKV, TuPV-1 viruses being the most frequently detected. On the other hand, TuNV, TuQV and NVU were the least widespread, found in just two libraries. Additionally, both BCMV and PV1 displayed high coverage on their genome and antigenome. This observation suggests that active viral replication is occurring within the mite, as single-stranded viruses produce a complementary strand in order to replicate [74]. The viral diversity of laboratory and field-collected mites did not show a clear difference. Interestingly, Belgian-derived samples in both conditions displayed similar outcomes in terms of the number of viral species. However, none of these viruses were found exclusively in Belgian samples; they were also widespread in samples from other countries. Conversely, two field-collected samples from Greece displayed only one viral species, TuPV-3, which, along with TuKV, is one of the most widespread viruses.

Overall, five viruses in this study were characterized within viral families known to infect plants, including *Potyviridae* [54], *Virgaviridae* [45], *Tombusviridae* [57], *Endornaviridae* [50], and *Kitaviridae* [75]. Mite transmission of viruses within these families has been reported [76,77,78,79], highlighting the importance of studying *T. urticae* as a potential phytopathogen transmitter. For instance, the presence of *Bean common mosaic virus* in *T. urticae* emphasizes the potential impact on bean agriculture, as the BCMV is known to cause severe yield losses and quality reduction in bean crops. The transmission of these viruses by *T. urticae* could exacerbate the challenges faced by farmers in managing viral diseases in beans, potentially leading to severe yield losses and quality reduction. Conversely, it could also improve crop quality production, which is a crucial source of dietary protein for millions of people worldwide.

The two-spotted spider mite, *T. urticae*, is a significant agricultural pest, feeding on about 1600 plant species and causing substantial economic losses to several crops. Our study explored the virome of *T. urticae*. Interestingly, the analysis of 14 libraries from field mite projects, compared with 16 libraries from other diverse projects, did not reveal significant differences. This observation may be attributed to variations in the geographical locations from which the control samples were obtained. This study reveals the potential plant pathogenic viral diversity associated with *T. urticae* and highlights the potential for this mite pest to transmit viral infections into various crops [80].

## Data Availability

The assembled sequences are currently under deposit at NCBI nucleotide database under submission ID: 2844176 and are also available at the Supplementary Table S3.

## Supplementary Materials

The following supporting information can be downloaded at https://www.mdpi.com/article/10.3390/v16101532/s1, Figure S1: *T. urticae* RNA-seq libraries acquisition flowchart; Figure S2: *Tetranychus urticae* virome characterization pipeline; Figure S3: Genome characteristics and conserved structures of characterized known viral sequences; Figure S4: RNA density plot; Table S1: Table summarizing essential information for all utilized libraries; Table S2: Table summarizing the outcomes of EVE characterization analyses; Table S3: Table summarizing the outcomes of BLASTN and BLASTX alignments for viral contigs, including details such as alignment subjects, percentage identity, and E-values; Table S4: Table presenting the transcripts per million TPM values.
