# Supplementary material for: Characterization of the Virome Associated with the Ubiquitous Two-Spotted Spider Mite, Tetranychus urticae
Source: Viruses. 2024 Sep 27;16(10):1532. doi: 10.3390/v16101532 (PMC11512250; doi:10.3390/v16101532)
Supplement: Supplementary file 1 [file viruses-16-01532-s001.zip › viruses-3216594_supplementary.pdf]

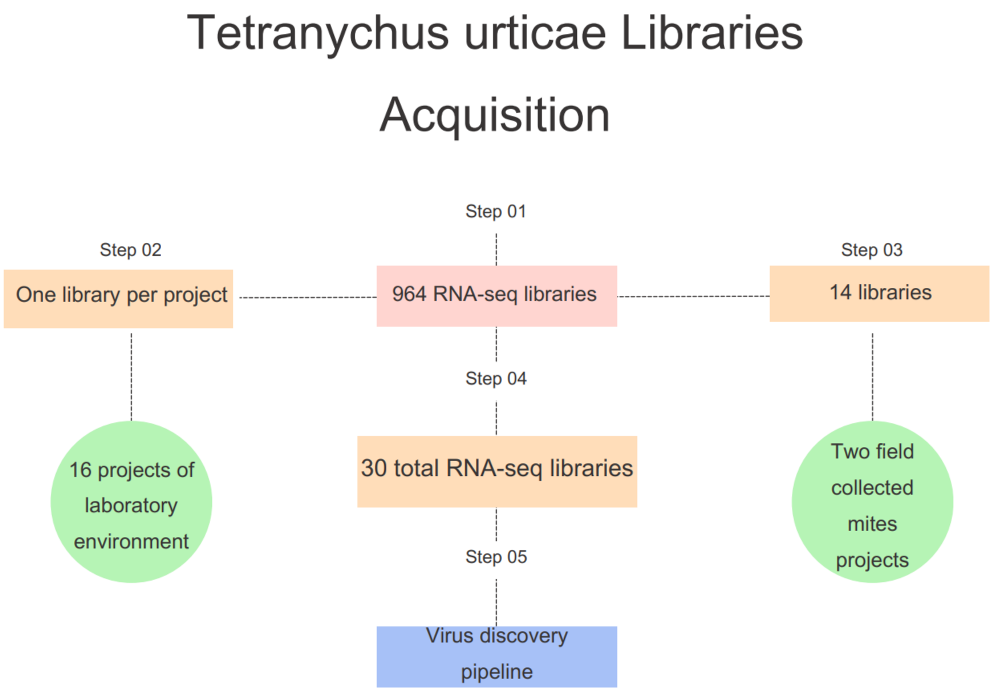

Figure S1. *T. urticae* RNA-seq libraries acquisition flowchart. All available RNA deep sequencing projects on the NCBI-SRA database were cataloged. From these, eighteen projects were selected: 16 libraries from mites collected in laboratory-controlled environments and 14 libraries from two projects with field-collected mites. All thirty RNA-seq SRAs were processed through the virus discovery pipeline in this study.

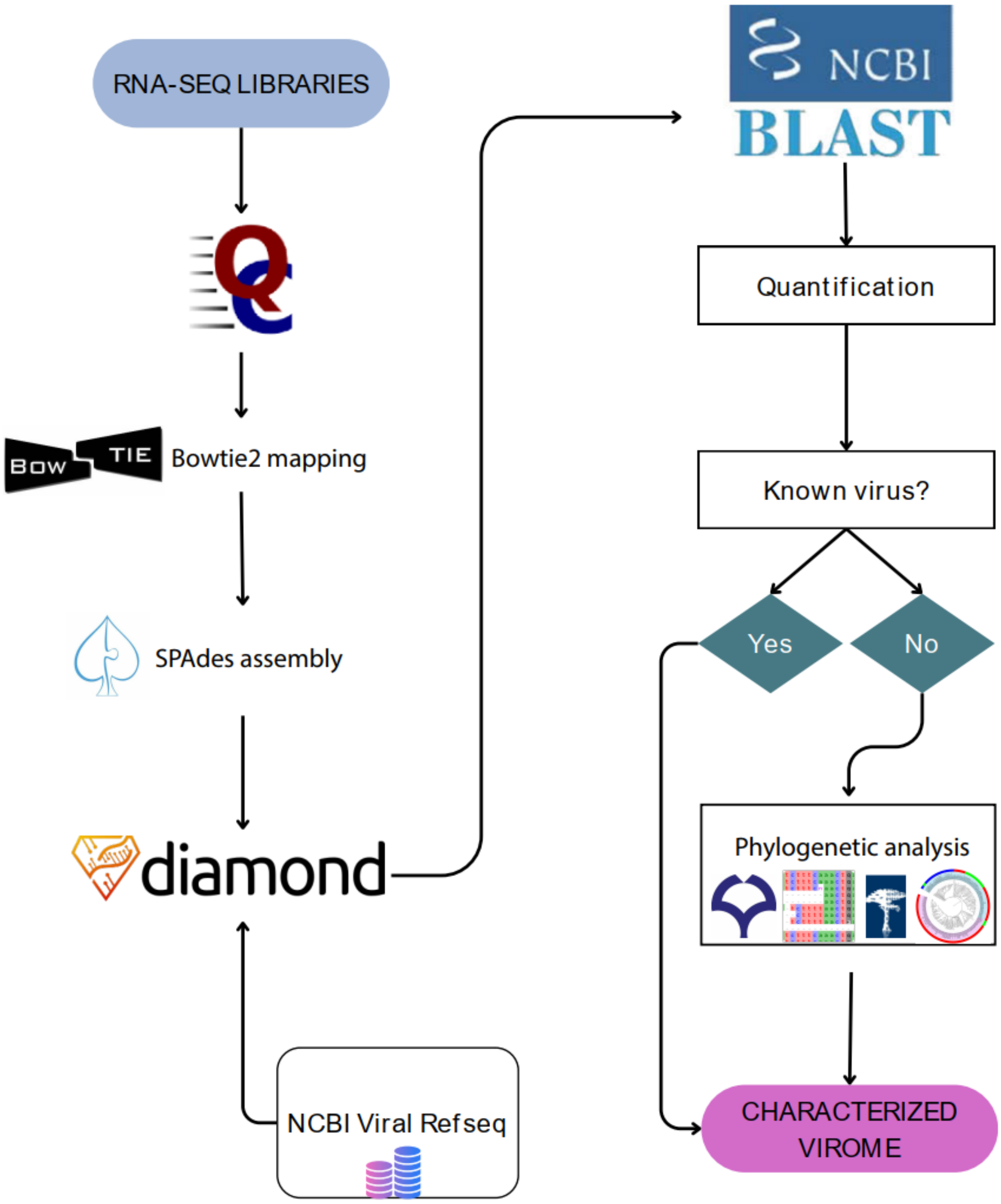

Figure S2. *Tetranychus urticae* virome characterization pipeline. Flowchart displaying the detailed pipeline used to characterize the virome associated with *Tetranychus urticae*. The process begins with the acquisition of RNA-seq libraries from the NCBI-SRA database. The selected libraries undergo quality control and filtering to remove low-quality reads and contaminants. Subsequently, bioinformatics tools are used to assemble the sequences. The assembled contigs are screened for viral sequences using BLAST searches. Identified viral sequences are then put through phylogenetic analysis.

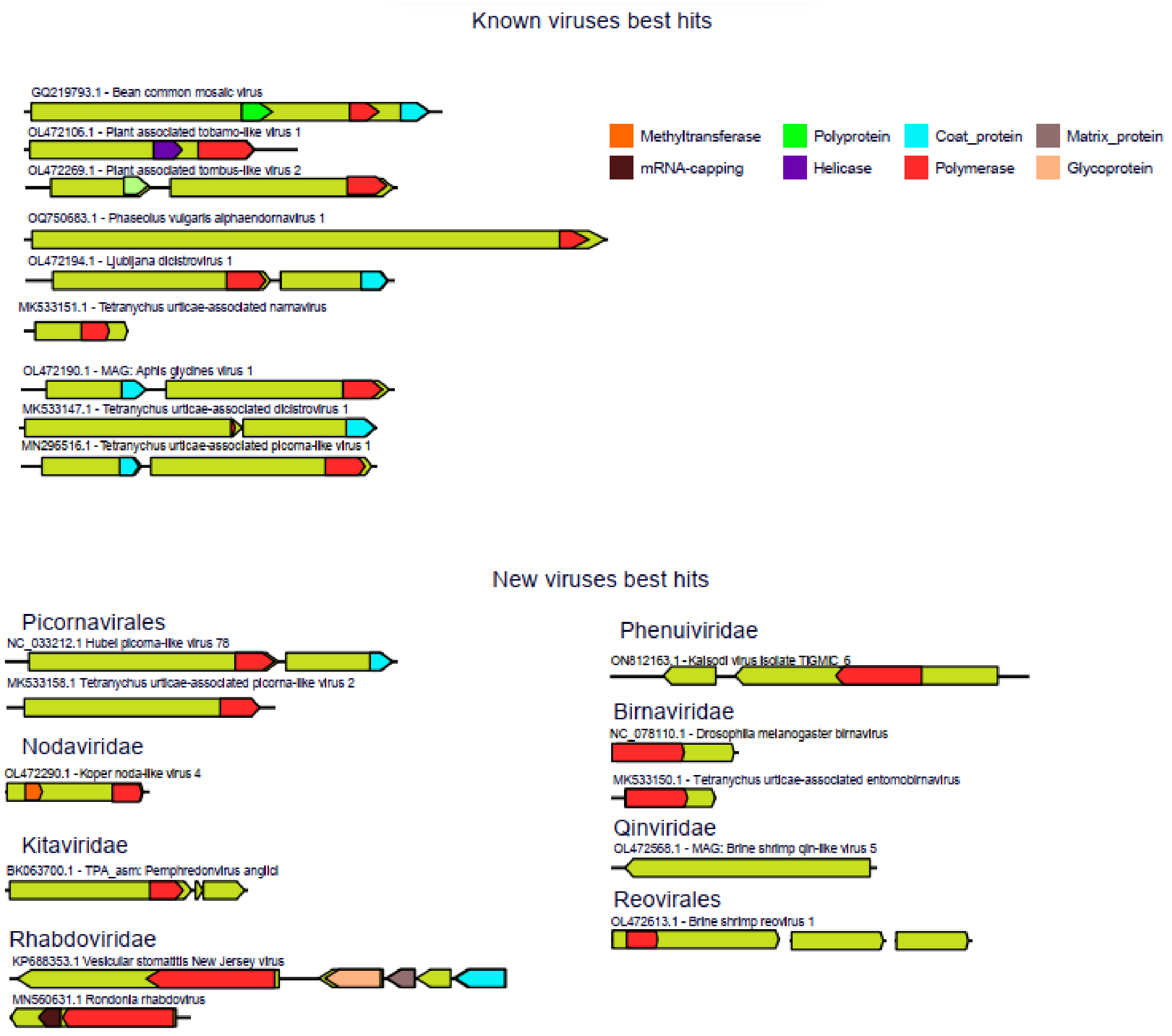

Figure S3. Genome characteristics and conserved structures of characterized viral sequences. Figure displays the open reading frames (ORFs) and conserved domains of characterized known viral sequences.

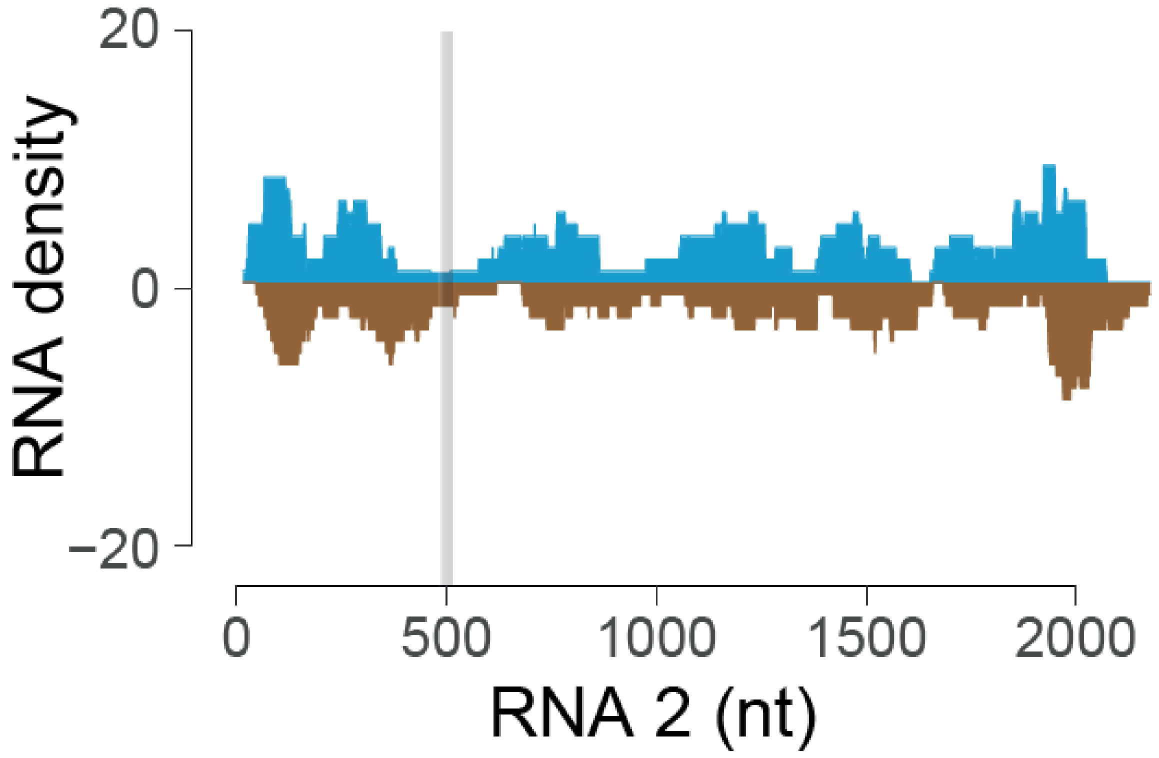

Figure S4. RNA density plot. RNA density plot of the viruses BCMV(A) and PV1(B). Positive sense strand is the depicted in blue and negative sense strand is depicted in brown.
